# MEDICARE AND THE DYNAMICS OF US HEALTH REFORM POLITICS

**DOI:** 10.1093/geroni/igac059.136

**Published:** 2022-12-20

**Authors:** Michael Gusmano, Alan Monheit

**Affiliations:** Leigh University, Bethlehem, Pennsylvania, United States; Rutgers University School of Public Health, Piscataway, Massachusetts, United States

## Abstract

Since the 2020 election, President Biden and Congressional Democrats have largely abandoned proposals for creating a “Medicare for All” system or adding a “public option” to the Affordable Care Act (ACA) marketplaces. This reflects a lack of sufficient enthusiasm, within the Congress or the public, for either option. These options are viewed by many current Medicare beneficiaries as a potential threat to their existing benefits. Indeed, framing the ACA and other efforts to expand insurance coverage as a threat to Medicare has become something that health reform opponents have done routinely since the 2010 midterm election. We will apply behavioral economics principles including loss aversion, hyperbolic discounting, and status quo bias, to explain why it has been easy to demonize reform options such as these. In addition to presenting data from public opinion polls, we will examine how Congressional and other party leaders have framed these choices for the public.

